# COVID-19, Vascular Diseases, and Vascular Services

**DOI:** 10.6061/clinics/2020/e1979

**Published:** 2020-05-18

**Authors:** Pedro Puech-Leão, Luiz Antonio Machado César, Nelson De Luccia

**Affiliations:** I Instituto Central (ICHC) e Instituto do Coracao (InCor), Hospital das Clinicas HCFMUSP, Faculdade de Medicina, Universidade de Sao Paulo, Sao Paulo, SP, BR.

COVID-19 is a respiratory disease that causes severe pneumonia and sepsis. It affects mainly the lungs, but its effects on other organs can reflect the severity of sepsis in a patient. Arterial and venous thrombosis have been noted in necropsy studies, suggesting a direct and/or indirect action of the virus on the endothelium and the coagulation cascade.

Endothelial involvement in bacterial and viral infections is not a new finding. It is known to be mediated by cytokines and has been shown to occur in pneumonia caused by influenza (1,2). In the case of influenza infection, the prevalence of myocardial infarction and associated mortality rates is higher among patients ≥65 years of age. The impact on mortality is alleviated by vaccine immunization. Influenza also affects the incidence of stroke in hypertensive patients. Myocarditis, mediated by cytokines caused by a direct effect of the virus on myocytes, has also been described.

Myocardial infarction and myocarditis have been described in 20-30% of patients hospitalized for COVID-19. They are associated with a significant increase in the risk of all-cause mortality (3). Stroke has also been reported as a result of large-vessel occlusion in young patients with COVID19 – one more aspect of the conundrum that is being studied during this pandemic. COVID-19 is more aggressive than previous viral respiratory diseases, and systemic manifestations are expected to be more severe. One study showed the presence of the virus in the vessel wall, indicating direct infection (4); this also has been seen in other infectious diseases. Disseminated intravascular coagulation (DIC) occurs in several different infectious diseases, especially those progressing to sepsis.

Deep venous thrombosis (DVT) is a frequent finding in intensive care unit (ICU) patients when investigated with Ultrasound (US) Doppler. The incidence of DVT is between 25% and 32% without prophylaxis and drops to between 10% to 18% when low-molecular-weight heparin is used (5). Most of these venous obstructions occur in calf veins and are clinically undetectable. They can cause pulmonary embolism (PE), often resulting in obstruction of small branches of the pulmonary arteries; however, many of these PEs are also clinically undetected, especially in patients with pneumonia on assisted ventilation.

New pathophysiology of COVID-19 cannot be established based on the observations of DIC, small artery thrombosis, or DVT in COVID-19. We may be facing a new viral pneumonia more aggressive than other previously known ones, with a more severe involvement of the vascular system.

No viral pneumonia has ever been studied with so much depth. Because of the worldwide impact of COVID19, the disease is being critically examined, including detailed autopsy examinations and laboratory testing. However, there are obstacles to assessing the vascular involvement of COVID-19. Doppler US is not feasible to examine these patients only for research purposes because of potential exposure of radiologists and technicians to the virus and potential virus spread through probes and other components of the US system that could act as fomites. Lung CT-scans for the diagnosis of COVID-19 are performed without contrast media, not permitting the evaluation of pulmonary arteries. The administration of contrast (for research purposes only) would be deleterious to the already affected kidney. Thus, only necropsy reports are available as valuable data, and these necropsies are likely being conducted in much detail than has ever been done in other viral infections.

There is an accumulation of data on the involvement of the vascular system in COVID-19, and new findings are expected to be revealed shortly. It is a new virus. We just have to be careful enough to not reinvent the wheel.

If, on one hand, uncertainties and controversies surround knowledge about the transmission mechanisms, epidemiological curves, immunity, pathophysiology, and treatment of COVID-19, on the other hand, a drastic change in the routine practice of vascular surgery, as a medical specialty, has been quite homogeneous in many countries and continents.

Working in a general hospital, with 900 beds transformed to serve exclusively patients affected by COVID-19, observing 200 intensive care unit beds being quickly filled by the uninterrupted arrival of new patients, and being obliged to transform surgical rooms into new ICU beds, what seemed to be a distant Asiatic epidemic has crystallized to a harsh and suffering reality.

Recognized as an unparalleled challenge by colleagues from the American and from the European Societies of Vascular Surgery, some who have lived and others who are still living in the epicenter of this terrible pandemic, with some days of delay we felt in Brazil the bitter taste of the same sanitary convulsion. São Paulo State, in which more than 20,000 diagnosed cases, has more than 1700 deaths attributed to the virus so far, compared to New York, Italy, and Spain. This has transformed the astonishment of those who considered the disease a distant occurrence into a sad and real experience (6).

Our entire hospital complex has around 2000 beds. With a coordinated administrative effort, it was possible to allocate non-COVID-19 patients in separate institutes, moving the vascular surgery ward. However, this allocation was followed by an immediate change in the surgical routine and suspension of all elective cases. As the specialty deals with emergency cases daily, such as ruptured or symptomatic aneurysms with imminent rupture, only these and other acute cases became part of a restricted number of operations that included severe cases of critical limb ischemia with a high risk of amputation or life-threatening infections. Curiously, due to mechanisms not yet thoroughly understood, even the volume of these emergencies has decreased when compared with the usual volume. More deaths may be occurring at home, due to patients’ fear of coming to the hospitals. This may be another cause of mortality, indirectly caused by the virus.

By separating infected and non-infected patients into separate quarters, a logical strategy of facing this pandemic and curbing this highly transmissible disease would be to avoid transit between quarters. However, this strategy will cause an extra problem in team management since this will lead to the creation of two populations of patients with vascular emergencies. Emergency staff rotation had to be organized, with the difficulty of personnel shortage. But, soon, special abilities of each specialty began to be jointly completed to fight the disease: the vascular teams assumed the care of the accesses and catheters for central venous monitoring or dialysis in the COVID ICUs. Thus, the complications of these procedures started to increase because they were frequently performed by less experienced teams.

The infection of team members is highly likely during this process, and protective equipment became mandatory, increasing tension and anxiety in the teams. Regular face-to-face meetings were replaced, when possible, by virtual meetings, bringing also significant changes to routine and relationships. Concerns with the teaching and specific surgical training caused anxiety to residents and fellows.

This moment is one of profound reflection. The humanitarian attitude displayed by residents as volunteers who have traveled to infected areas, comforting patients and families, can compensate the negative aspects this disease can cause and can open the possibility for personal growth in these trainees

The pandemic is still growing, but our knowledge of it also continues to improve. The outlook is optimistic considering that some patients have already been cured of it and a vast amount of knowledge has been gathered so far. Also, young doctors are incorporating this priceless experience into their personal and professional growth.

This phrase can convey a little hope during these difficult times:

“Today, man, in order to be saved, needs only one thing: to open his heart to joy” – Bertrand Russell

## AUTHOR CONTRIBUTIONS

All of the authors conceived the study and were responsible for the manuscript drafting, editing and review.
